# r-tPA with loading dose of clopidogrel and aspirin therapies for capsular warning syndrome attributed to middle cerebral artery atherosclerotic stenosis: A CARE-compliant case report

**DOI:** 10.1097/MD.0000000000019247

**Published:** 2020-02-28

**Authors:** Jinghan Xu, Meiting Zhuang, Guanshui Bao, Yu Zhai, Guo-Yuan Yang, Gang Xue, Qiang Li

**Affiliations:** a Department of Neurology, Shanghai Ninth People's Hospital, Shanghai Jiao Tong University School of Medicine; b Neuroscience and Neuroengineering Research Center, Med-X Research Institute and School of Biomedical Engineering, Shanghai Jiao Tong University; c Department of Neurology, Fengcheng Hospital of Fengxian District, Shanghai; d Department of Neurology, The First Affiliated Hospital of Soochow University, Suzhou, China.

**Keywords:** capsular warning syndrome, dual anti-platelet, intracranial atherosclerotic stenosis, intravenous thrombolysis, recombinant tissue plasminogen

## Abstract

**Rationale:**

The capsular warning syndrome (CWS) is a rare and special type of transient ischemic attacks (TIAs) syndrome. The pathophysiology of CWS is very complicate, and intracranial atherosclerotic stenosis (ICAS) is rare cause. Moreover, the effective and standard therapy has not yet been established.

**Patient concerns:**

A 47-year-old man experienced repeated and exacerbated TIAs of right hemiparesis and dysarthria. Fourteen hours after the first episode of TIAs, he developed more severe right hemiparesis and dysarthria, the National Institute of Health Stroke Scale (NIHSS) score was 12 points, and did not recover in a long time.

**Diagnosis:**

The computed tomography (CT) angiography displayed high stenosis in the M1 segment of the left middle cerebral artery. The patient was diagnosed as CWS with ICAS.

**Interventions:**

Loading dose of clopidogrel and aspirin were started but were ineffective, then we used recombinant tissue plasminogen (r-tPA) for thrombolysis therapy after repeat CT scan that showed small acute infarcts in the right putamen and no bleeding.

**Outcomes:**

The patient was successfully treated by r-tPA intravenous thrombolysis after loading dose of dual-anti-platelet. He recovered rapidly, and the NIHSS score was 0 point, modified Rankin Scale score was 0 point, and Barthel Index score was 100 points at 3-month follow-up.

**Lessons:**

r-tPA combined with loading dose of dual antiplatelet appears safe and effective in carefully selected CWS patients with ICAS. The collection of similar cases and further randomized controlled trial research would be desirable.

## 1 Introduction

The capsular warning syndrome (CWS) is a rare and special type of transient ischemic attacks (TIAs) syndrome with a recurrent stereotypical motor or sensory deficits (such as aphasia, aversion, neglect) and no cortical signs.^[1]^ The pathophysiology of CWS has been suggested, such as hemodynamic impairment, microembolism from artery to artery or from the heart, vasospasm.^[2]^ Only few cases reported CWS was attributed to large artery atherosclerotic stenosis.^[1]^ About 40% to 60% of CWS patients finally developed cerebral infarction within 10 days, but effective and standard therapy has not yet been established.^[1,2]^ Here we reported a CWS patient attributed to middle cerebral artery (MCA) atherosclerotic stenosis, and the patient was treated by recombinant tissue plasminogen (r-tPA) intravenous thrombolysis after loading dose of dual antiplatelet with a good clinical outcome.

## 2 Case presentation

**Figure 1:**
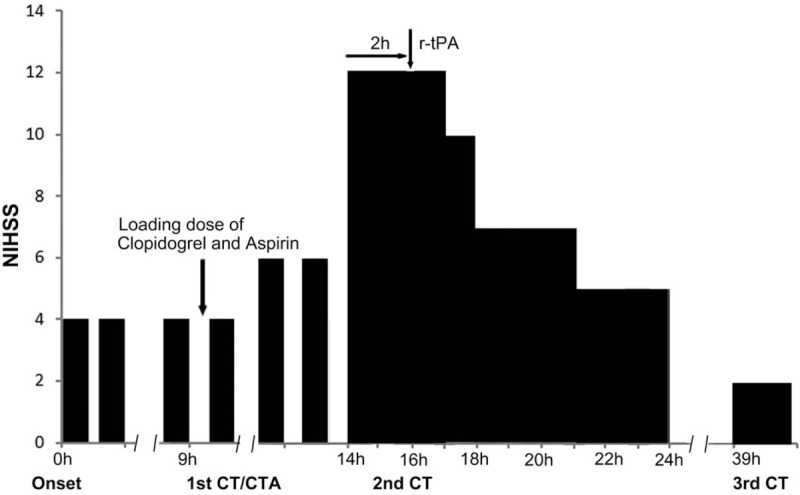
The clinical course of the patient. The horizontal bar shows the time course (hour); the vertical bar shows the National Institute of Health Stroke Scale (NIHSS) score. CT = computed tomography, CTA = computed tomography angiography, NIHSS = National Institute of Health Stroke Scale, r-tPA = recombinant tissue plasminogen.

**Figure 2:**
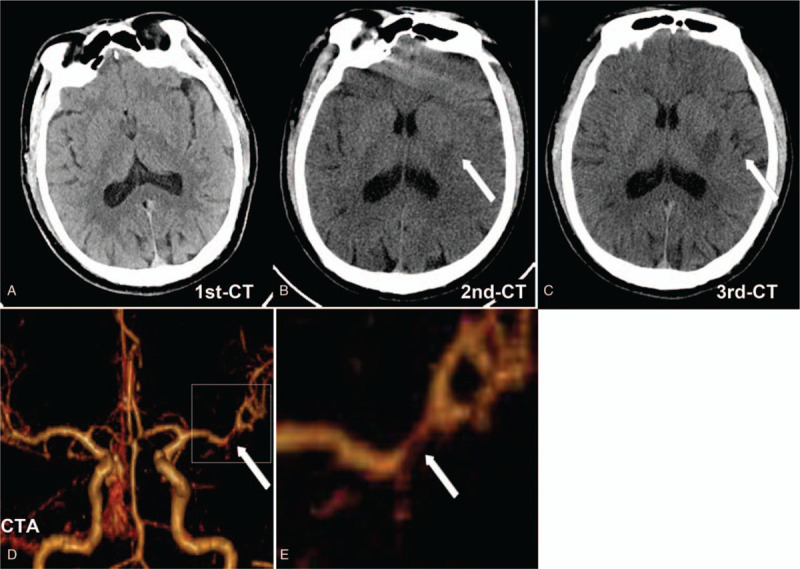
Brain CT and CTA of the patient. (A) Brain CT at 5 hours after the first episode of TIAs (the third TIAs) shows normal. (B) Brain CT at 14 hours after the first episode of TIAs (repeat CT before r-tPA thrombolysis) shows small acute infarcts in the right putamen (white arrow). (C) Brain CT at 39 hours after the first episode of TIAs (24 hours post r-tPA thrombolysis) shows larger acute infarcts in the right putamen (white arrow) and no bleeding. (D) Brain CTA shows M1 segment stenosis of the left middle cerebral artery (white arrow). (E) The higher magnification of M1 segment stenosis(white arrow, Fig.2 E is the boxed area in Fig. 2D). CT = computed tomography, CTA = computed tomography angiography, r-tPA = recombinant tissue plasminogen, TIAs = transient ischemic attacks.

A 47-year-old man with hypertension suddenly developed right hemiparesis and dysarthria at 9 o’clock in the morning, and his symptoms disappeared within a few minutes. He had a long history of smoking and hypertension and was treated with amlodipine 5 mg once a day in the past 2 years. He experienced hemiparesis and dysarthria attack once again and also rapidly recovered. He had a third stereotypical episode at around 4 PM, and then was taken to our hospital immediately. He has completely recovered when arriving at the emergency department and the National Institute of Health Stroke Scale (NIHSS) score was 0 point. No abnormalities such as bleeding or tumor were found in the examination of brain computed tomography (CT) scan. We treated him with clopidogrel 300 mg and aspirin tablets 300 mg immediately. Another 3 similar attacks occurred for 5 hours after administration of loading dose of the dual antiplatelet therapy, and the attack duration extended longer time (Fig. 1). Fourteen hours after the first episode of TIAs, he developed more severe right hemiparesis and dysarthria, and the NIHSS score was 12 points (Fig. 1). The computed tomography angiography (CTA) results showed that M1 segment of the left MCA was highly stenosis, but the distal end of the M2 segment was well clear (Fig. 2). Repeat CT scan showed small acute infarcts in the right putamen and no bleeding. The loading dose of dual antiplatelet treatments seemed to be ineffective in the acute phase, and the patient declined to proceed for digital subtraction angiography. We administered r-tPA (46.8 mg, 0.6 mg/kg) at 76 minutes after final unrecovered attack of right hemiparesis and dysarthria. His NIHSS score immediately decreased to 10 points at 1 hour after r-tPA administration and further decreased to 2 points at 24 hours post r-tPA administration. Brain CT at 24 hours post r-tPA thrombolysis shows larger acute infarcts in the right putamen and no bleeding, and then we continued aspirin, clopidogrel, and statins treatment. He gradually recovered, the NIHSS score was 0 point, modified Rankin Scale score was 0 point, and Barthel Index score was 100 points at 3-month follow-up.

## 3 Discussion

The prevalence of intracranial atherosclerotic stenosis (ICAS) is higher in Oriental and Asian populations than in Western populations, but there are few studies on the treatment and mechanism of ICAS in patients with CWS. This study is the first to report a CWS patient attributed to ICAS treated with r-tPA thrombolysis and loading dose of dual antiplatelet with the good clinical effect.

As regards the mechanisms of CWS, the atherosclerotic stenosis of penetrating artery has been widely accepted by most researchers at present.^[3,4]^ The lenticulostriate arteries originating from the MCA are the most commonly affected artery in CWS. Although multiple studies have confirmed the penetrating artery mechanism of CWS,^[3,4]^ the pathophysiology of large vascular atherosclerosis has not yet been fully elucidated. The perfusion deficits of large intracranial artery may lead to CWS, but the case is rare. Donnan et al's^[1]^ study reported only 1 patient with ipsilateral MCA stenosis among 50 CWS patients, which was caused by the stenosis of the left MCA and the hypoperfusion of the penetrating artery area. In this case, the M1 segment stenosis of the left MCA leads to the hypoperfusion and the formation of thrombosis which blocks the origin of the penetrating artery, which results in recurrent TIAs. The pathogenesis of CWS caused by ICAS is mainly due to the obstruction of the proximal portions of penetrating artery and the microembolism from artery to artery.

The lack of effective and standard therapy for CWS is a potential dilemma to the future study, especially for the CWS patient with ICAS. Several reports have showed loading dose of dual antiplatelet therapy for CWS patients had a good clinical effect. Kawano et al^[5]^ reported that a loading dose of clopidogrel was given for CWS patient when the 10th episode appeared after initially treating with aspirin, argatroban, and cilostazol, and finally the symptoms did not recur. Asil et al^[6]^ developed a loading dose of double antiplatelet strategy (clopidogrel and aspirin 300 mg each) for 2 patients with CWS, and the symptoms were significantly prevented and the frequent TIAs episodes were terminated. In previously reported cases of CWS, the recurring episode was stabilized with plural antiplatelet therapy.^[7]^ When clopidogrel is started at a conventional doses, it takes several days to get their antiplatelet effects, while a loading dose of them can reach effective levels within 2 hours after administration.^[8]^ So we started loading dose of dual antiplatelet therapy (clopidogrel 300 mg + aspirin 300 mg) at the early stage for the patient. However, initially loading dual antiplatelet therapy was not effective in our case. For the first time, we gave an extra intravenous r-tPA therapy after loading dose of dual antiplatelet for CWS with ICAS.

The most crucial goal is the prevention of infarction for CWS. The case showed that progressive symptoms were unresponsive to loading dose of dual antiplatelet. Theoretically, r-tPA is effective for not only dissolving fibrin thrombus in the stenosis region of MCA but also microthrombus at the origin of the lenticulostriate artery. Vivanco-Hidalgo et al^[9]^ treated 4 CWS patients with intravenous thrombolysis, and 3 of them made a full recovery with no lesion presented in DWI. Camps-Renom et al^[10]^ reported that 8 of 12 patients with r-tPA had a good prognosis. There was a case of CWS without ICAS that was successfully treated with r-tPA and other antithrombotic therapies.^[11]^ However, there were also some discrepancies in the current reports of IV r-tPA therapy for CWS patient. Tassi et al^[12]^ reported that intravenous thrombolysis did not prevent the evolution of CWS, and didn’t demonstrate higher efficacy than other treatments at 3-month follow-up. All reports confirmed that r-tPA thrombolysis for CWS patients did not significantly increase the probability of any symptomatic intracranial hemorrhages. In our case, the pathogenesis of CWS was associated with highly atherosclerotic stenosis of MCA. This patient suffered the frequent and extended TIAs duration, and has a high risk of cerebral infarctions. We supposed r-tPA may be an effective and safe treatment in our case. Our result also actually showed r-tPA was effective and safe on CWS patient with ICAS. This is the first case of CWS patient attributed to atherosclerotic stenosis of intracranial artery, who was treated with loading dose of dual antiplatelet combination with r-tPA.

The presence of left MCA atherosclerotic stenosis was confirmed by CTA diagnosis, but endovascular intervention therapy was not performed because of informed consent problem. Lee et al^[13]^ reported a case of CWS patient with MCA stenosis, who got a favorable prognosis after receiving intravascular intervention treatment to improve the perfusion of the perforating artery. However, we recommend to early r-tPA combined with loading dose of dual antiplatelet treatment for CWS patients with ICAS in a primary hospital lack of neurointervention condition. Furthermore, previous CWS thrombolysis cases showed that the prognosis was closely related to r-tPA treatment time window.^[7,11,12,14]^ The time window always was delayed because r-tPA thrombolysis usually was used in the patients with high-frequency TIAs, long duration, and high NIHSS score at onset. Therefore, CWS patients who have 3 times TIAs episodes or any ischemic symptoms lasting more than 30 minutes, should be evaluated according to the acute ischemic stroke guidelines.

## 4 Conclusion

CWS with ICAS is rare, and this case suggests r-tPA combined with loading dose of dual antiplatelet appears safe and effective on carefully selected CWS patients with ICAS. The collection of similar cases and further randomized controlled trial research would be desirable.

## Author contributions

**Conceptualization:** Gang Xue, Qiang Li.

**Data curation:** Jinghan Xu, Meiting Zhuang.

**Funding acquisition:** Qiang Li.

**Investigation:** Jinghan Xu, Guanshui Bao.

**Project administration:** Gang Xue, Qiang Li.

**Resources:** Qiang Li.

**Software:** Guanshui Bao.

**Supervision:** Gang Xue, Qiang Li.

**Validation:** Gang Xue, Qiang Li.

**Visualization:** Jinghan Xu.

**Writing – original draft:** Jinghan Xu, Meiting Zhuang.

**Writing – review & editing:** Yu Zhai, Guo-Yuan Yang, Qiang Li.

Qiang Li orcid: 0000-0003-1350-4008.
